# Simulated Design–Build–Test–Learn Cycles for Consistent Comparison of Machine Learning Methods in Metabolic Engineering

**DOI:** 10.1021/acssynbio.3c00186

**Published:** 2023-08-24

**Authors:** Paul van Lent, Joep Schmitz, Thomas Abeel

**Affiliations:** † Delft Bioinformatics Lab, Delft University of Technology Van Mourik, Delft 2628 XE, The Netherlands; ‡ Department of Science and Research, Joep Schmitz - dsm-firmenich, Science & Research, P.O. Box 1, 2600 MA Delft, The Netherlands; § Infectious Disease and Microbiome Program, Broad Institute of MIT and Harvard, Cambridge, Massachusetts 02142, United States

**Keywords:** combinatorial pathway optimization, machine learning, DBTL cycles, metabolic engineering, automated recommendation

## Abstract

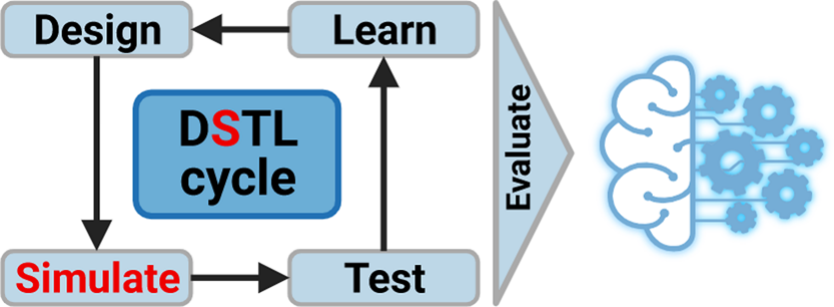

Combinatorial pathway optimization is an important tool in metabolic flux optimization. Simultaneous optimization of a large number of pathway genes often leads to combinatorial explosions. Strain optimization is therefore often performed using iterative design–build–test–learn (DBTL) cycles. The aim of these cycles is to develop a product strain iteratively, every time incorporating learning from the previous cycle. Machine learning methods provide a potentially powerful tool to learn from data and propose new designs for the next DBTL cycle. However, due to the lack of a framework for consistently testing the performance of machine learning methods over multiple DBTL cycles, evaluating the effectiveness of these methods remains a challenge. In this work, we propose a mechanistic kinetic model-based framework to test and optimize machine learning for iterative combinatorial pathway optimization. Using this framework, we show that gradient boosting and random forest models outperform the other tested methods in the low-data regime. We demonstrate that these methods are robust for training set biases and experimental noise. Finally, we introduce an algorithm for recommending new designs using machine learning model predictions. We show that when the number of strains to be built is limited, starting with a large initial DBTL cycle is favorable over building the same number of strains for every cycle.

## Introduction

Metabolic engineering focuses on optimizing microorganisms through genetic interventions in metabolic pathways, intending to increase the flux toward a product of interest.^1,2^ Classically, metabolic engineering applies sequential debottlenecking of rate-limiting steps in a pathway of interest.^3,4^ While this method has shown success in many metabolic engineering problems, fundamental limitations to this procedure exist. Despite the vast work on characterizing metabolic networks, there is still a substantial lack of knowledge about many metabolic pathways, especially the regulation of individual pathway elements and cell physiology. As a result, purely rational engineering of pathways remains challenging.^5−7^ On top of that, sequential optimization of pathways potentially misses the global optimum configuration of pathway elements (*e.g.*, enzyme concentrations) that maximize the product flux.^8^

**Figure 1:**
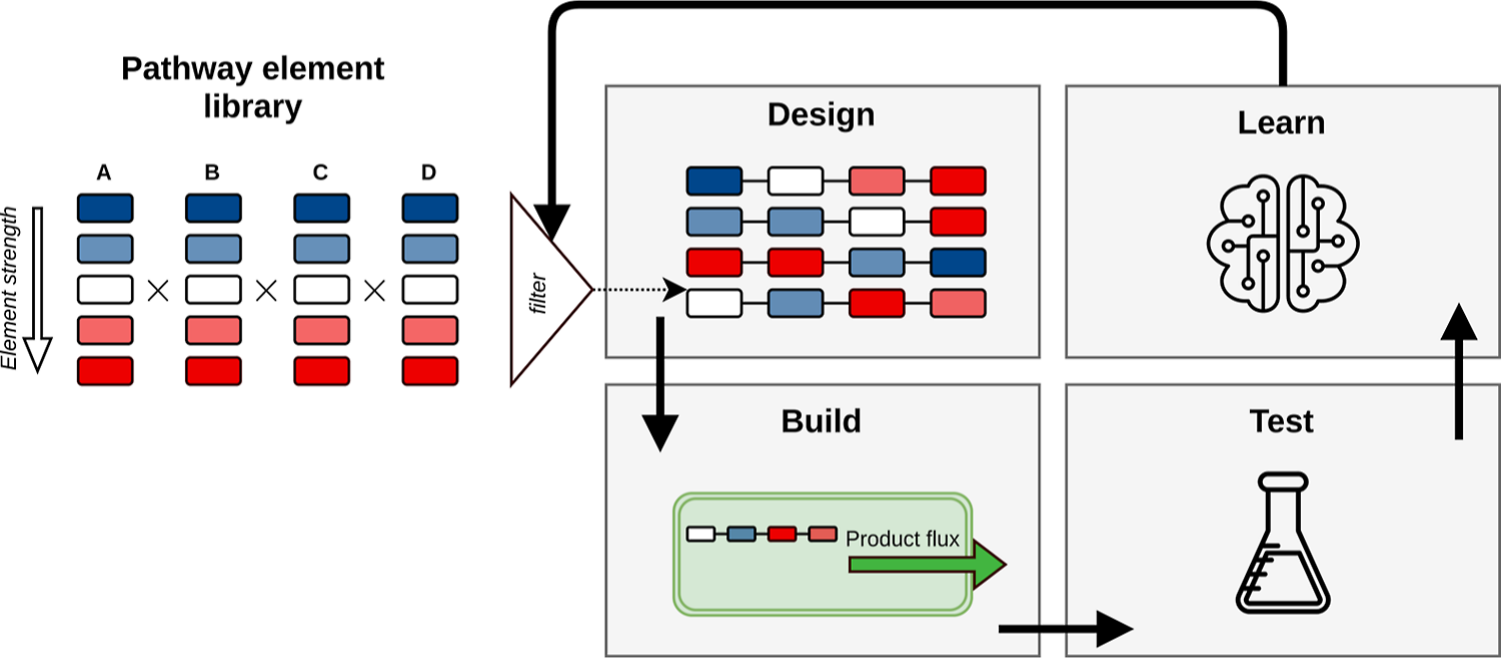
Design–build–test–learn (DBTL) cycle in metabolic engineering. In combinatorial pathway optimization, designs are chosen from a large DNA library with pathway elements: promoters, RBSs, CDSs, and other elements that might influence a protein concentration or catalysis rate. Due to the combinatorial explosion of the design space, only a small subset of these designs is built and tested experimentally (filter step). From these experiments, important properties for engineering are ideally learned and used to design a new set of strains to be built. By iterating this process, the pathway is optimized until the strain is industrially relevant.

Recent progress in synthetic biology, genome engineering, and high-throughput building and screening of microbial strains now allows for targeting multiple pathway components simultaneously, paving the way for combinatorial pathway optimization.^8^ The major advantage of this approach is that there is a reduced chance of missing the optimum pathway configuration, and many successes have been reported for increasing the titer/yield/rate (TYR) values of products using this strategy.^9−13^ In combinatorial pathway optimization, strain designs are constructed from a large DNA library consisting of promoters, ribosomal binding sites, coding sequences, and other DNA components that have an effect on enzyme properties or concentrations. These designs are assembled and introduced into a microorganism.^14^ Due to the large set of library components, a combinatorial explosion of the design space often occurs, making it experimentally infeasible to test every design. Combinatorial pathway optimization is therefore often performed in an iterative fashion using design–build–test–learn (DBTL) cycles (Figure 1).^11^ The idea is to build an initial set of strain designs and use the generated data from the test phase to learn important characteristics of the pathway. This information is then used to guide engineering in the next cycle. DBTL cycles for microbial strain development are widely adopted. Nevertheless, developing an economically feasible bioprocess is still considered very costly and time-consuming. Strategies on how to find the best-producing strain with as little experimental effort as necessary remain an open question. Furthermore, the limited availability of public data for multiple DBTL cycles is complicating a systematic comparison of different strategies for their performance.^15^

Machine learning has been increasingly used for guiding engineering.^1^ For metabolic flux optimization, this ranges from identifying the targets for engineering through unsupervised learning^16^ to predicting metabolite concentrations from proteomics data using supervised learning.^17^ Another potential application of machine learning is for recommending new strain designs for the next DBTL cycle by learning from a small set of experimentally probed input designs, which would allow (semi)-automated iterative metabolic engineering.^18−21^ One example is the automated recommendation tool, which uses an ensemble of machine learning models to create a predictive distribution, from which it samples new designs for the next DBTL cycle given a user-specified exploration/exploitation parameter.^20^ While the automated recommendation tool was successfully applied to optimize the production of dodecanol and tryptophan, instances where the method did not perform well were also reported.^20^ This might be attributed to the complexity of the pathways and the lack of data from more than two DBTL cycles.^20,22^ More fundamentally, a framework for the comparison of machine learning methods and optimization strategies of the integrated DBTL workflow over multiple iterations is lacking.

Much effort has been put into understanding and modeling cellular metabolism.^23−25^ We propose to use a mechanistic kinetic model-based framework for the optimization and application of machine learning methods in iterative metabolic engineering. In kinetic modeling, changes in intracellular metabolite concentrations over time are described by ordinary differential equations (ODEs). Each reaction flux is described by a kinetic mechanism that can, in principle, be derived from the laws of mass action, so that kinetic parameters can directly be interpreted as biologically relevant quantities. This property of kinetic models allows for *in silico* changes in the properties of a pathway element, such as increasing the enzyme concentration or changing the catalytic properties of an enzyme.^23,26,27^ The ODE model is used to simulate data for comparing the performance of machine learning methods. We start by addressing which machine learning algorithms are favored for this particular use case and test the effect of training set biases and measurement noise on the predictive performance. Building on these results, a recommendation algorithm is introduced to automate DBTL cycles. We demonstrate how our framework can be used to optimize strain development workflows over multiple DBTL cycles for different DBTL cycle strategies.

## Results and Discussion

Publicly available data sets of multiple cycles are scarce due to the costly and time-consuming nature of these experiments.^18,20,22,28^ This complicates the validation and comparison of machine learning methods as well as the comparison of DBTL cycle strategies. For example, the validation of recommendation algorithms requires that separate DBTL cycles are performed for the same metabolic pathway problem. Similarly, testing multiple DBTL cycle strategies for the same metabolic pathway would realistically never be performed due to the costly nature of these experiments. Simulated data offer an advantage over real-world data as many of these practical limitations are overcome. We will first explain how the kinetic model-based framework was developed to represent a metabolic pathway embedded in a model of cell physiology.

### Representation of a Metabolic Pathway Using a Kinetic Modeling Approach

To test different metabolic pathway topologies with distinct thermodynamic properties, we integrate a synthetic pathway into a previously established *Escherichia coli* core kinetic model that was implemented in the symbolic kinetic models in Python (SKiMpy) package.^29,30^ The aim of this study is not necessarily to build the best possible kinetic model of a specific pathway. Instead, we aim to provide a generic representation of a hypothetical pathway that captures pathway behavior (*e.g.*, enzyme kinetics, topology, and rate-limiting steps) and is embedded in a physiologically relevant cell and bioprocess model.^10,20,28^

**Figure 2:**
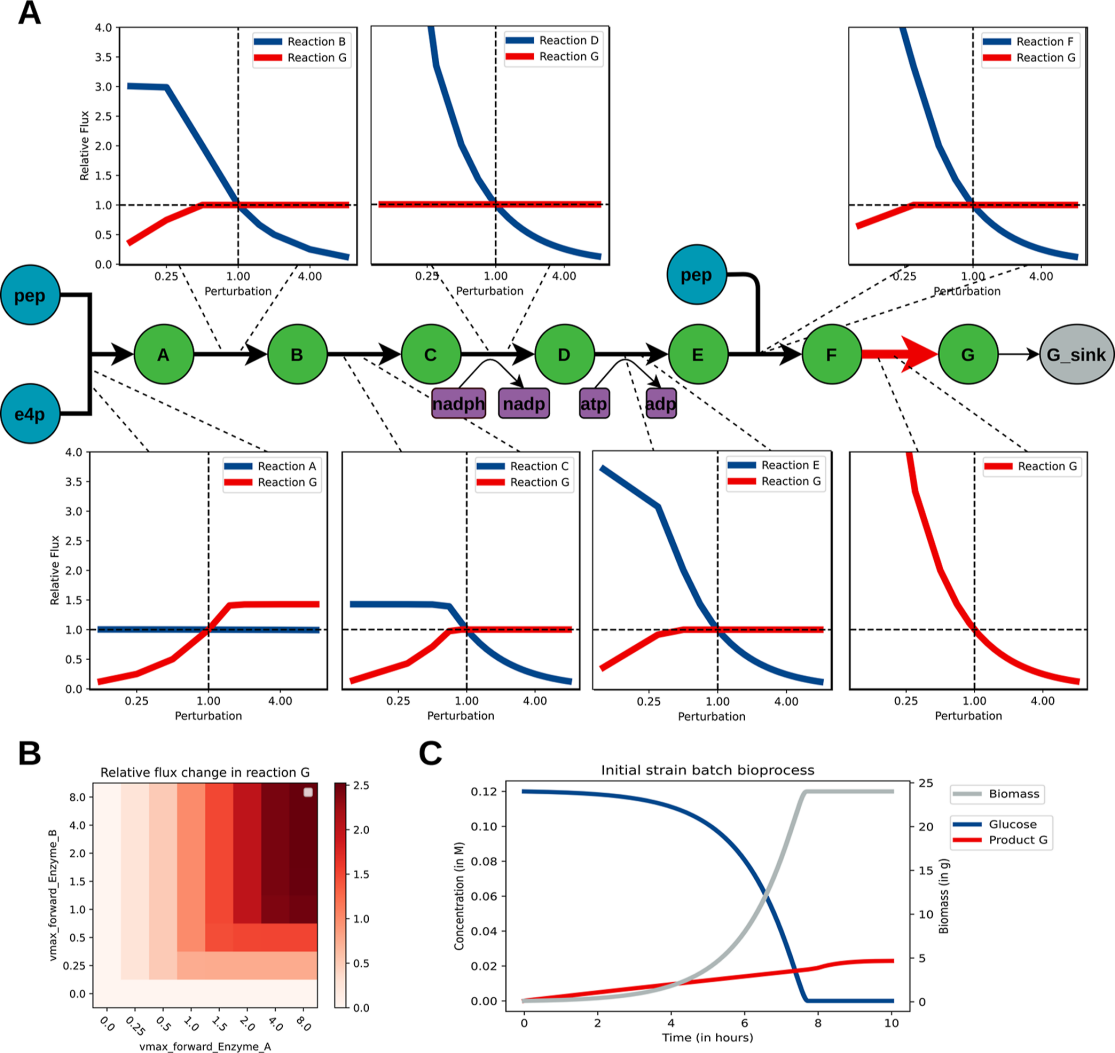
Local enzyme concentration perturbations result in changes in flux. (A) Simplified schematic of the integrated pathway. The pathway is coupled to the core metabolism through the substrates erythrose-4-phosphate (e4p) and phosphoenolpyruvate (pep), as well as the cofactors nadph and atp. For each reaction, response in the steady-state flux through reaction G and its respective reaction is shown, where local enzyme perturbations are performed on the range [0.125,8]. Perturbations are defined with respect to the enzyme concentrations of the initial strain (*i.e.*, ). (B) Example of a combinatorial design space for reactions A and B. Note that the behavior of perturbing enzymes A and B is not obvious from the local perturbation shown in A. (C) Batch bioprocess of the initial (heterologous) strain with the implemented pathway. Although this model was constructed for intracellular metabolism, a simple batch bioprocess can be modeled, with depletion of glucose, biomass growth, and product formation.

A schematic representation of the pathway is shown in Figure 2A. A degradation reaction was added as a boundary condition, and the optimization objective is to maximize the production of compound G. The response of the product flux G to perturbations of the enzyme concentrations are shown as panels along the pathway (Figure 2A). Despite the pathway being almost linear, the influence of enzyme concentrations on this flux is nonintuitive. For example, perturbations of enzyme A do not lead to differences in its respective reaction flux but do lead to a 1.5-fold increase in the product flux (Figure 2A, lower left panel). Another example is the response to local perturbations in enzyme B. Here, a decreasing reaction flux is observed due to depletion of the substrate. However, no significant effect is observed on the product flux (Figure 2A, upper left panel). Another observation is that lowering the enzyme concentration in the last step (reaction G) of the pathway leads to an increase in net production (Figure 2A, lower right panel). Thus, increasing enzyme concentrations of the individual reactions do not lead to higher fluxes but instead lead to a decrease in flux due to depletion of reaction substrates. These observations stress the importance of combinatorial optimization of pathways as sequential optimization strategies might have nonintuitive outcomes.^8,17,31^ To illustrate this, the combinatorial optimization of reactions A and B is shown (Figure 2B). Note that increasing both enzyme concentrations leads to higher product flux compared to their individual local perturbations. We therefore conclude that the dynamics of the pathway captures the features of a typical pathway subject to metabolic engineering well (Figure 2B).

On top of being able to represent a metabolic pathway, the cell model should be embedded in a basic bioprocess model. Figure 2C shows the modeling of a 1 L batch reactor bioprocess, inoculated with 1 g of initial biomass.^30^ Glucose is consumed until it is depleted and an exponential biomass growth phase is observed. Furthermore, a product is formed in the process. After depletion of glucose, no more biomass is formed and the growth rate is zero. We show that this kinetic model captures important characteristics of a batch bioprocess. This could be extended to model other types of bioprocesses, such as fed-batch fermentation.^32^ While the implemented pathway considered here has no metabolic burden on the host, these types of effects could be captured by explicitly modeling the inhibitory effects of pathway intermediates on the biomass equation (see Figure S10).^7^ When including these types of interactions, as well as other types of pathways into the kinetic model using ORACLE sampling, the physiological relevance of the kinetic parameter sets can be easily verified.^30,33^

### Combinatorial Pathway Optimization Strategies Can Be Simulated and Used for Benchmarking Machine Learning Models

Although machine learning is increasingly being explored for combinatorial pathway optimization, it remains unclear how to optimize the approach over multiple DBTL cycles.^1,18,20,21^ For example, questions on the amount of strains that need to be built for effective learning, the effect of biases in the DNA library distributions on predictive performance, and how the DBTL cycle strategy should be set up over multiple cycles remain unsettled.

**Figure 3:**
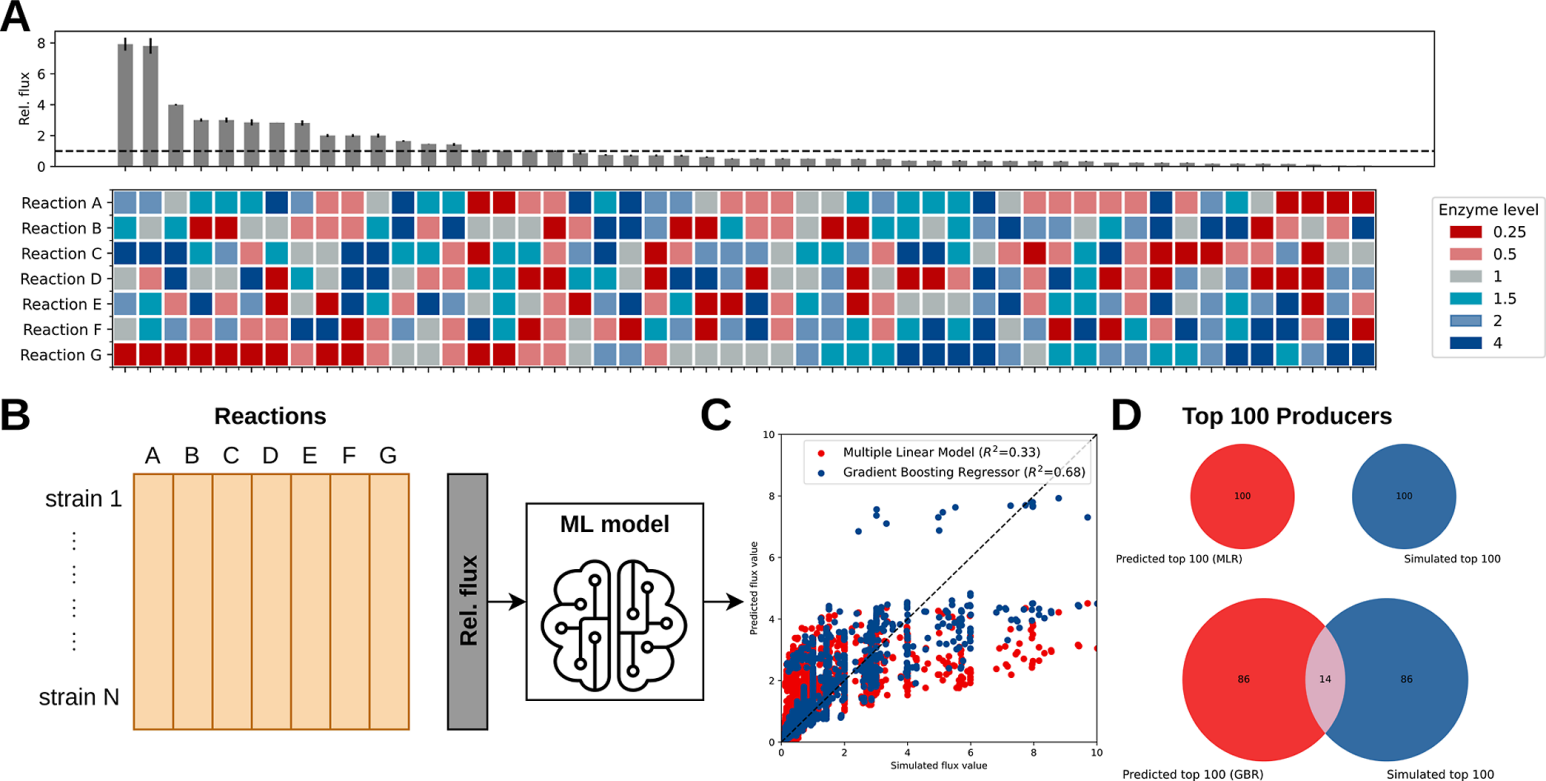
Example of the simulation of large strain libraries. (A) Example of 50 simulated combinatorial designs, where each enzyme level was chosen with equal probability. These levels could be achieved with a promoter from the DNA library. The distribution of the enzyme levels of each strain design is shown below the response curve of the metabolic flux through enzyme G. (B) The design space along with the relative flux increase can be used to train a predictive model. (C) Predicted *versus* simulated relative fluxes for a multiple linear regression model and a GBR, with *R*^2^ values of 0.33 and 0.68, respectively. (D) Venn diagrams of the performance of predicting the top 100 designs for the full combinatorial design space compared to the simulated combinatorial space (279.936 designs).

Figure 3A shows an example of a simulated metabolic engineering scenario for 50 designs, where enzyme levels are varied with respect to the enzyme level of the initial strain. The effect of adjusting enzyme levels was implemented in the model by changing the *V*_max_ parameters (see Methods). We assume here that the enzyme level change can be achieved with a set of DNA elements (*e.g.*, promoters or ribosomal binding sites) from a predefined DNA library. Considerable effort has been put into experimentally quantifying the promoter strength of promoter sequences,^34−36^ as well as predictive tools that guide promoter sequence engineering to achieve desirable enzyme expression levels.^37−40^ While in this study, five different enzyme levels were considered, this might not always be attainable in real-world metabolic engineering, due to the lack of promoters for a particular enzyme or not being able to achieve the up-/downregulation levels considered here. This challenge could be overcome in simulation studies by considering lower enzyme levels or considering enzyme levels closer to levels of the initial strain.

It can be observed from the strain designs that the highest-producing strains have a bias in low promoter strengths for enzyme G and tends to have upregulation of enzyme A (Figure 3A). In combinatorial pathway optimization, it generally occurs that some pathway steps are more important to increase product flux than others, while some have limited sensitivity. We therefore showcase that the simulated data captures key features of combinatorial optimization in real-world data.^10,41^ While in this work, only changes in enzyme levels were considered, other enzyme properties (*e.g.*, catalytic properties of the reaction) could be assembled in a similar fashion. This is especially important when the cost of expressing enzymes of a nonnative pathway needs to be considered.

The encoded designs along with the simulated product flux can then be used to train a model and compare based on two metrics (Figure 3B). These designs will be used to train different types of models. As an example, a multiple linear model and a nonlinear gradient-boosting regressor (GBR) are evaluated using two metrics. The Pearson correlation (*R*^2^) between the predicted *versus* simulated product flux gives a general impression of the predictive performance of both methods (Figure 3C). We use the intersection between the predicted top 100 and the simulated top 100 designs as an additional performance metric. This metric captures how well the model has learned the best-performing strains (Figure 3D). Together, these metrics will be used to consistently evaluate the effect of training set sizes, sampling scenarios, and noise models on the predictive performance of machine learning models. Here, the predictive performance of models is only evaluated on the flux through reaction G. When considering other objectives, such as biomass growth or reduction of toxic intermediate concentration in a pathway, models could also be compared for their performance as a multi-objective optimization problem.^42^ This can, for example, be done by defining the target variables as a weighted function of the objectives (see Figures S9, S10).

### Ensemble Methods Excel in the Low-Data Regime

**Figure 4:**
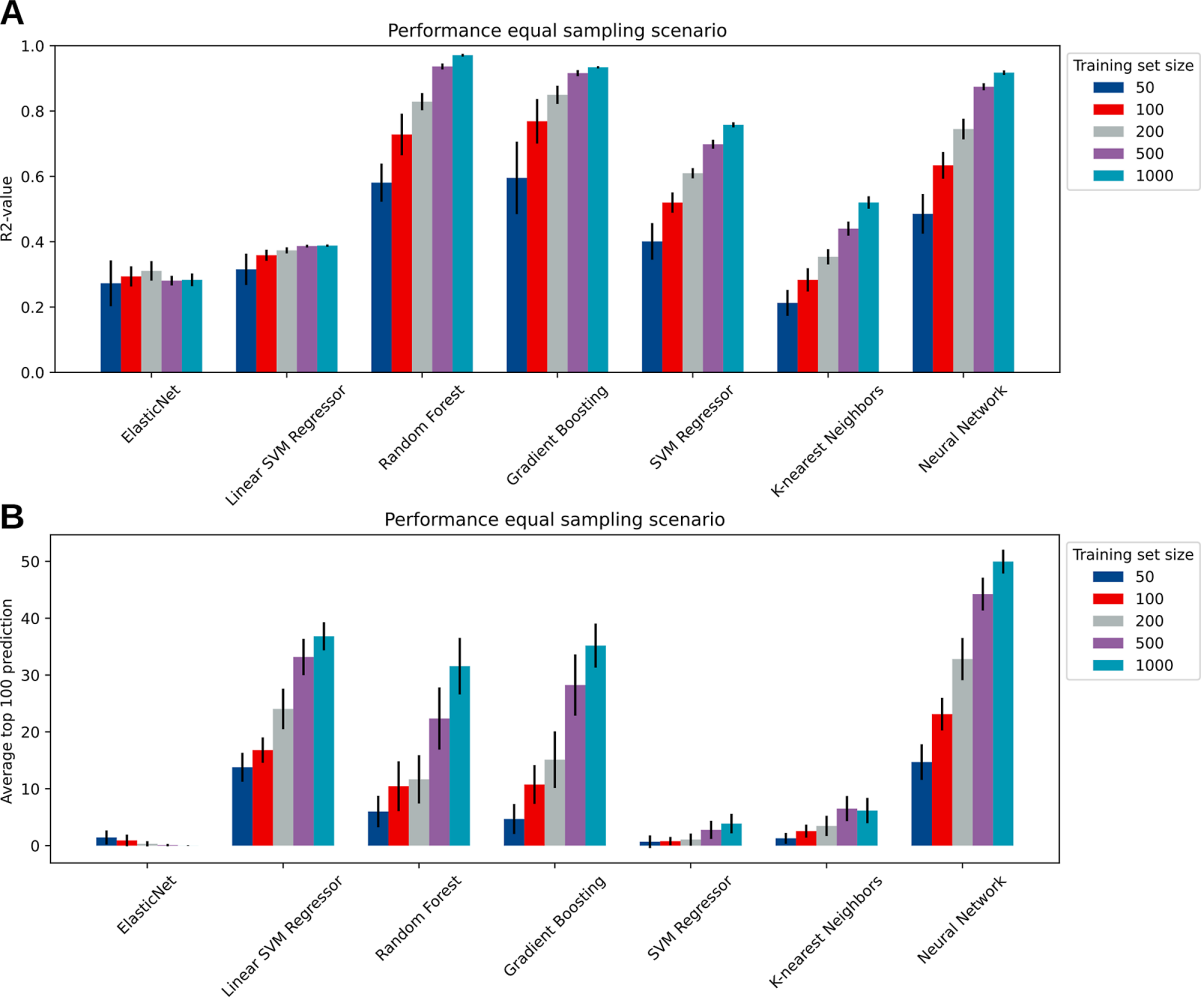
Influence of training set size on predictive performance. Seven machine learning algorithms (two linear and five nonlinear) were tested for their performance on two metrics: a general prediction on the combinatorial design space (*R*^2^ value), and the performance in pointing out the top 100 best-performing strains (predicted *vs* simulated). (A) *R*^2^ value over the full design space for the seven algorithms with increasing training set size (20 runs per training set size). (B) Intersection value between predicted *vs* simulated top 100 (averaged predictions, 20 runs per training set size).

Due to a large number of existing machine learning models, we sought to filter out the best-performing algorithms from an initial search using default parameters. Eight machine learning models are compared for different training set sizes, where each enzyme level was chosen with equal probability (see Methods). The product flux was predicted for the full combinatorial design space (Figure 4A,B).

Figure 4A shows the general performance of the machine learning models for increasing the number of data points in the training set. Overall, the nonlinear methods outperformed the linear methods for all training set sizes. This could be attributed to the observed nonlinear behavior of the pathway (Figure 2A, *all panels*). The ensemble methods of random forest and gradient-boosting trees outperform the other methods, specifically in the regime where training set sizes are small. This finding is consistent with a previous report that suggests that XGBoost (a regularized gradient-boosting algorithm) performs well when data are scarce.^21^ When training sets are larger, neural networks also tend to perform well. Due to the nature of the metabolic engineering experiments, methods that perform well and are stable on small training sets are preferred.

Results on the top 100 prediction metrics further support the view that ensemble methods and neural networks outperform other methods (Figure 4B). Neural networks here even outperform the ensemble methods. Interestingly, while the linear support vector machine regressor performs poorly on the general prediction task, prediction of the top 100 designs seems to be similar to, if not better than, the ensemble models (Figure 4B). We observed that predicting the top 100 best producers with the linear support vector machine indeed outperformed the ensemble methods, but plateaus in its predictive performance for very large training set sizes (see Supporting Information Figure S1). Based on these results, we concluded that the ensemble methods of gradient boosting and random forest, along with linear support vector machine and neural networks should be further investigated for their performance.

### Machine Learning Models Are Robust to Training Set Biases and Noise

**Figure 5:**
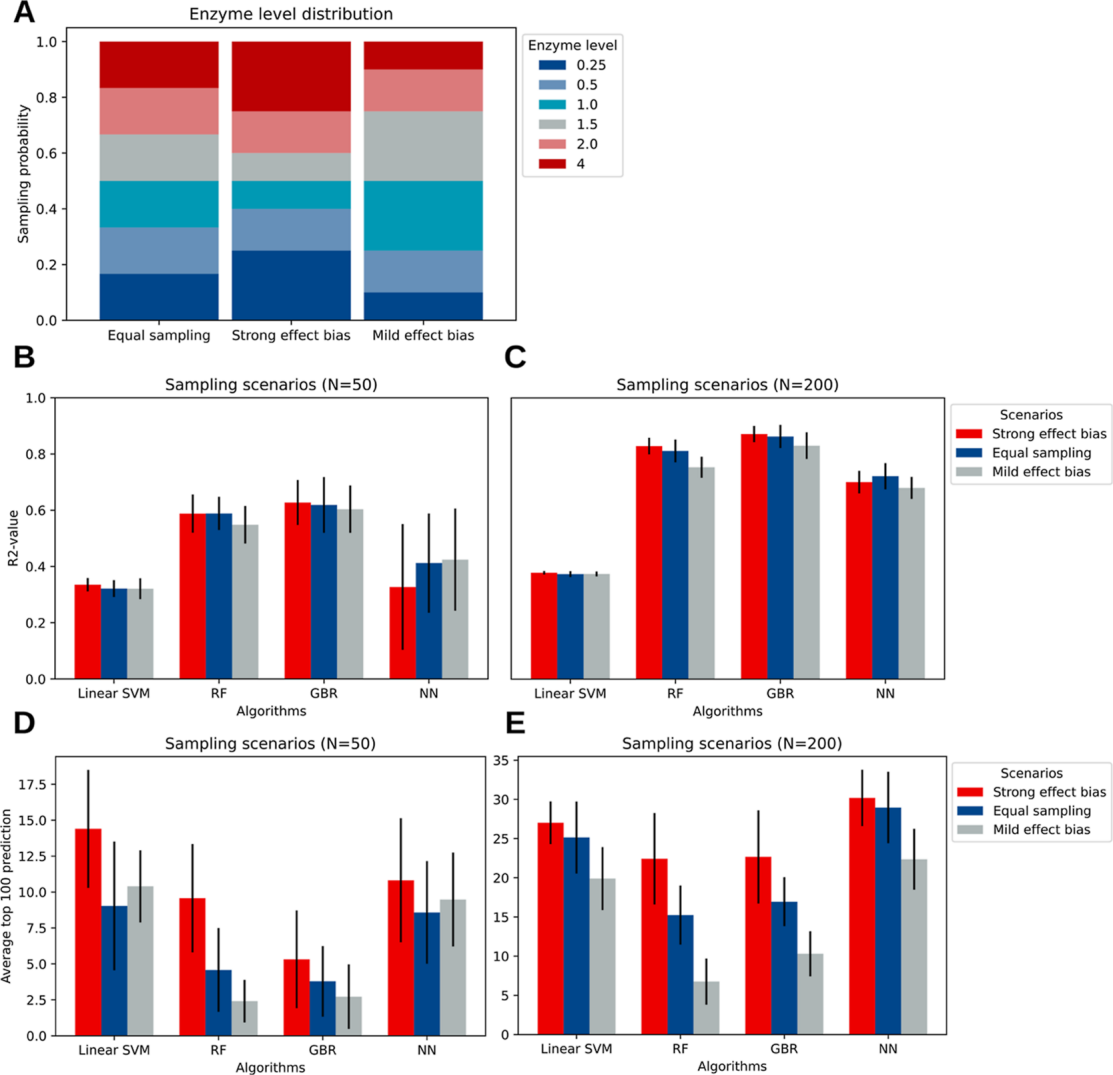
Effect of sampling biases on the predictive performance: (A) Three different sampling scenarios are defined (see Methods). The equal sampling scenario is unbiased, while the strong effect bias and mild effect bias are biased toward certain enzyme levels. (B,C) *R*^2^ for the four best-performing algorithms trained on 50 and 200 samples, respectively. (D,E) Top 100 prediction performance for the four best-performing algorithms trained on 50 and 200 samples, respectively. A slightly better performance is observed for the strong effect bias sampling scenario, which can be attributed to the fact that the strong enzyme levels with respect to the wild-type are overrepresented in the simulated top 100.

An important aspect for ensuring good generalization of predictions to other unseen designs is that the initially built strains are a proper representation of the combinatorial design space. In many combinatorial pathway optimization procedures, these first strains are often built using a design-of-experiment approach, such as full factorial or fractional-factorial design setups.^10,43−45^ Alternative to design-of-experiment approaches, designs can be assembled in a probabilistic fashion through the use of DNA libraries.^22^ Even though this might not lead to an optimal set of designs (see Figure S7), it allows for building many strains in a one-pot transformation approach. However, this method potentially introduces biases in the distribution of the assembled designs. To understand the effect of enzyme-level distribution biases of designs on the model performance, we determined three scenarios: a bias for higher enzyme expression levels (strong effect bias), a bias that has enzyme expression levels closer to the initial strain values (mild effect bias), and a scenario where all enzyme levels are equally represented (equal sampling) (Figure 5A).

The general prediction performance (*R*^2^) and the top 100 predictions after Bayesian hyperparameter optimization are shown for two different training set sizes (*N* = 50 and *N* = 200) (Figure 4B–E). For the general prediction task, we do not observe a significant difference in performance between the three scenarios, indicating that there is only a marginal effect of the sampling distribution on how well the design space is learned for all algorithms (Figure 4B,C). For the top 100 prediction task, a difference is observed, as the scenario with biases toward stronger enzyme levels tends to have higher predictive performance (Figure 4C,D). This effect can largely be attributed to the optimization problem that is considered here. The top 100 simulated strains mostly consist of enzyme levels that have a strong perturbation effect with respect to the initial strain, which leads to a slightly better performance of the *strong effect bias* distribution. Due to the marginal effect of the enzyme-level distributions on the *R*^2^, we expect that this does not generalize to other pathways.

In addition to testing the influence of enzyme-level distribution biases, we also tested the influence of noise on the predictive performance. A homoscedastic and heteroscedastic noise model was used on the measured product flux for two different noise percentages (4 and 15%), but no significant effect was found for the four models considered above (Figure S2 and Tables S3 and S4).

### DBTL Cycle Simulations Reveal Experimental Design Principles That May Guide Real-World Metabolic Engineering Experiments

While in certain cases, the optimal design can immediately be predicted from the initial sampling (*i.e.*, the first DBTL cycle), other cases require multiple DBTL cycles to increase product fluxes to a desirable level. The challenge of effectively suggesting new strain designs for the next DBTL cycle using machine learning remains challenging. Several recommendation algorithms have been introduced in the literature,^19−21^ among which the automated recommendation tool (ART) stands out as a notable example.^20^ While a rigorous benchmark of the different recommendation algorithms is outside the scope of this study, an example of how simulated DBTL cycles can be used for this purpose is reported in the Supporting Information (see Figure S8).

**Figure 6:**
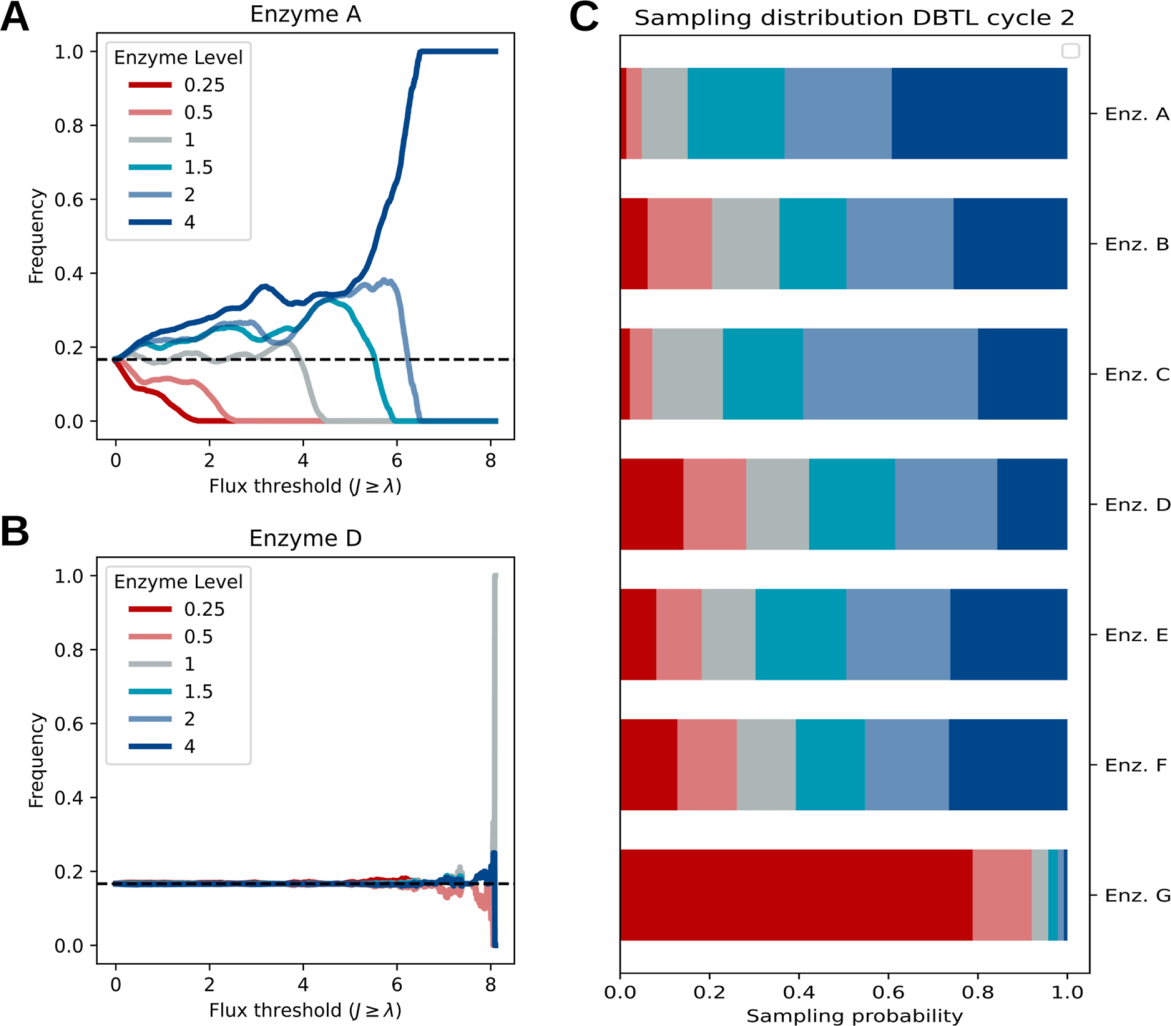
Recommending new designs based on the learned combinatorial space. For each enzyme, the frequency of each enzyme level is calculated (promoter strength) as a function of the threshold. Then, we take the AUC of all enzyme levels and normalize. (A,C) Example of the frequency for enzyme A when a model is trained on 200 samples. Higher enzyme levels are more frequent than the lower levels for increasing predicted production of a strain. When taking the AUC, this is also observed in the sampling distribution of promoters for the next round. (B,C) Example of a feature that is not considered important for the optimization problem. As the threshold increases, we do not see a certain enzyme level (promoter strength) being favored, which can also be observed from the sampling distribution.

To test the behavior of ML algorithms over multiple DBTL cycles, we introduce a fast and model-free recommendation algorithm to the automated recommendation. Learned from an initial sampling scenario using any machine learning algorithm, the predicted design space is used to generate a sampling distribution of the enzyme levels for each enzyme (see Methods). In short, frequencies of enzyme levels for each enzyme are counted above a certain flux threshold (J ≥ λ*). The frequencies of the enzyme levels in the subspace can then be followed as a function of the threshold λ* (Figure 6). To go from this threshold plot to a sampling distribution, the area under the curve (AUC) is taken and normalized for the enzyme level of the respective enzyme.

Two enzymes are shown as an example of how the sampling distribution is generated from the learned combinatorial design space. The GBR is trained on 200 training samples, but we note that this recommendation algorithm is independent of the used model. We observe from the threshold frequency plots that enzyme A has a bias toward higher enzyme levels in the space where the flux is high. After taking the AUC, it is observed that the new sampling distribution captures this bias, which means that in the next DBTL cycle, it is more likely that the higher enzyme levels are chosen (Figure 6A,C). In contrast, enzyme D does not seem to have any bias toward certain enzyme levels as a function of the threshold, with the frequencies being close to equiprobability even in the higher flux range. This leads to an almost equal sampling distribution for enzyme D in the next DBTL cycle (Figure 6B,C). From an experimental perspective, the biased enzyme-level distributions can be achieved by adjusting the DNA content in the library transformation. DNA sequencing can then be used to confirm whether there was a successful introduction of the intended bias.^46^ Using this approach, it is now possible to completely automate DBTL cycles and use this to test different DBTL cycle scenarios (see Figures S3–S6).

We utilize the model-free recommendation algorithm to evaluate the performance of DBTL cycle scenarios under different settings. These settings may include variations in the number of samples per round, utilization of different models, *etc*. Here, we showcase three distinct DBTL cycle strategies. For all three scenarios, only 250 strains are allowed to be built over the course of all DBTL cycle rounds: every round building a similar number of strains, a large set of strains in the first cycle and then a decreasing number of strains, and starting with only a few strains and building many strains in the later stages (see Methods and Table 3). Every scenario is initialized by an equal sampling scenario, as described above (see Figure 5).

Table 1 reports the performance of the three DBTL cycle scenarios for the GBR, as this algorithm was shown to outperform the other methods (see Table S5). For all scenarios, we do not observe significant differences in the performance of the strategies after all DBTL rounds have been performed. One interesting observation is that the scenario with a large initial building phase already performs well on the top 100 predictions and does not improve drastically over the rounds. This indicates that for some metabolic engineering problems, DBTL cycles are not necessarily required to optimize strains as long as your initial sampling is large. In these cases, the design of the next DBTL cycle should increase the size of the design space. This could be extending the range of the considered enzyme levels (*e.g.*, by considering strong promoters) or using other pathway elements that were not included in the initial DNA library transformation.

**Table 1:** Performance of Three DBTL Cycle Scenarios for the GBR^a^

strategy	metric	cycle 1	cycle 2	cycle 3	cycle 4	cycle 5
(50,50,50,50,50)	*R*^2^	0.47 ± 0.15	0.74 ± 0.07	0.81 ± 0.05	0.84 ± 0.04	0.87 ± 0.03
	top 100 prediction	43.97	62.27	72.7	80.03	82.67
(150,50,25,25)	*R*^2^	0.75 ± 0.07	0.82 ± 0.05	0.84 ± 0.04	0.84 ± 0.03	
	top 100 prediction	78.9	82.03	84.3	83.1	
(25,25,50,150)	*R*^2^	0.32 ± 0.19	0.58 ± 0.07	0.78 ± 0.05	0.87 ± 0.03	
	top 100 prediction	16.6	38.9	59.84	83.9	

a For all five cycles, the predicted top 100 and *R*^2^ are reported.

To conclude, we have developed a framework for a consistent comparison of machine learning methods over multiple DBTL cycles. We show that the GBR outperforms other methods in the low-data regime. After introducing a recommendation algorithm, we show that for this metabolic flux optimization problem, multiple DBTL cycles are not always necessary when the first cycle has many built strains. The developed framework is not limited to this pathway, but can also be extended to other pathways with distinct topologies. Furthermore, strain-building methods other than the DNA library transformations considered here (*e.g.*, design-of-experiment approach) could be compared using this framework, similar to how we tested the effect of training set biases (Figures 5, S7).^10,43−45^

## Methods

**Figure 7:**
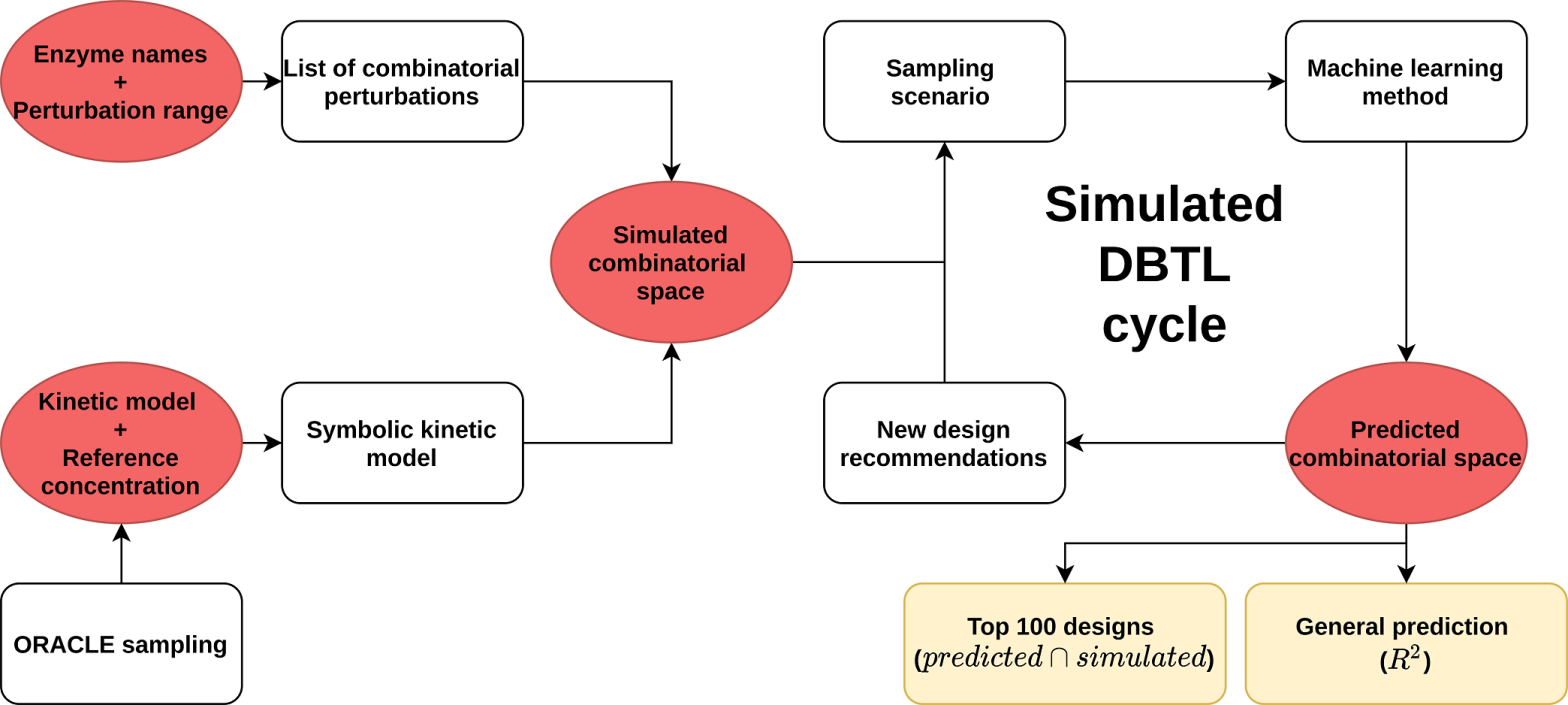
Overview of the pipeline used in this study. A synthetic pathway is integrated into the kinetic model to be used for ODE simulations. From this space, we test multiple sampling scenarios and train seven models. Two different performance metrics are used for the evaluation. As the simulation of the full design space is computationally intensive, the intermediate files of the pipeline are saved (shown in red).

An overview of the simulation pipeline used in this study is shown (Figure 7). The input kinetic model with the included synthetic pathway is constructed as described in the next section. Enzyme names and perturbation values are used to construct a combinatorial design list to simulate steady-state flux rates compared with respect to the wild-type. In the first DBTL cycle round, an initial sampling scenario is chosen to create training sets. Noise is then added to the target value (product flux) based on a noise model to mimic experimental/technical noise. Next, a machine learning model is trained, and the full combinatorial design space is predicted. The performance is measured based on two metrics: the general predictive performance described by the *R*^2^ and the performance in predicting the top 100 designs by comparing it to the simulated top 100 designs. All steps were performed in Python 3.9 and all codes used in this study are available at https://github.com/AbeelLab/simulated-dbtl.

### Including Synthetic Pathways in an *E. coli* Core Kinetic Model

We use a previously published kinetic model of the core metabolism of *E. coli* that was reformulated to be used in the SKiMpy package.^29,30^ SKiMpy is a recently developed Python package for semi-automated generation of kinetic models, along with a variety of tools for downstream analysis.^30^ The model consists of 64 metabolites involved in 65 reactions, of which 49 metabolites have an ODE. The other metabolites are considered constant boundary conditions and are not directly involved in the pathway that was implemented.

We wish to include a pathway with thermodynamic properties that is reminiscent of a biological pathway into the core metabolism of *E. coli*. With this in mind, we use the structural thermokinetic modeling framework to generate kinetic models that are consistent with stoichiometric and thermodynamic constraints, as well as a biomass optimization objective often used in constraint-based models.^47−49^ We start by adding a pathway to the stoichiometry of the core model and add thermodynamic information from a provided database to impose a flux directionality profile.^48,50,51^ Additional constraints on the steady-state metabolite concentrations of the pathway are imposed (see the Supporting Information). Based on these constraints, the biomass objective that was provided is optimized, and a sample of fluxes, concentrations, and equilibrium constants is taken from the feasible solution space. Next, from the reaction stoichiometry and thermodynamic properties, the kinetic mechanisms were identified that are available in SKiMpy.^30^ Finally, we back-calculate and sample kinetic parameters from the fluxes, concentrations, and kinetic mechanisms and check whether they lead to stable behavior of the ODE system using ORACLE parameter sampling.^52^ Additional details on the stoichiometry and constraints are further provided below and in the Supporting Information (see Table S1).

### Included Synthetic Pathway

A seven-reaction pathway that was inspired by the shikimate pathway is included, with phosphoenolpyruvate and erythrose-4-phosphate as substrates (Table 2).^53^ Metabolite G is considered as a boundary condition that is kept constant. The optimization objective is the maximization of the flux through reaction G. Thermodynamic information was added from a thermodynamic database that was included in the pyTFA package.^50^ Further information on the kinetic parameters is provided in the Supporting Information (see Table S2).

**Table 2:** Overview of the Properties of the Synthetic Pathway^a^

reaction	name	d*G* (in kJ/mol)	kinetic mechanism
pep + *e*4*p* → *A* + pi	reaction A	–12.03	Irrev. MM
*A* → *B*	reaction B	–16.12	Irrev. MM
*B* ↔ *C*	reaction C	–8.47	Rev. MM
*C* + nadph ↔ D + nadp	reaction D	1.10	Gen. Rev. Hill
*D* + atp → *E* + adp	reaction E	–6.40	Gen. Rev. Hill
pep + *E* ↔ *F* + pi	reaction F	0.41	Gen. Rev. Hill
*F* → *G* + pi	reaction G	–11.77	Irrev. MM

a Newly included metabolites are capitalized, while the already modeled metabolites are shown with lowercase letters. Thermodynamic flux analysis was performed using the pyTFA package to add additional constraints on the feasible parameter space for sampling steady-state consistent parameter sets.^50,52^ The kinetic mechanisms used are Irreversible Michaelis Menten (Irrev. MM), Reversible Michaelis Menten (Rev. MM), and Generalized Reversible Hill (Gen. Rev. Hill). Kinetic parameters for each reaction are reported in the Supporting Information.

### Metabolic Engineering Scenarios

The goal is to generate synthetic metabolomics data from the previously described kinetic model for scenarios that are realistic in industrial metabolic engineering.^10,11,22^ We therefore perturb the *V*_max_ of a particular enzyme in the pathway, solve the initial value problem of the ODE system until it reaches a steady state, and calculate the relative increase/decrease of the target flux with respect to the wild-type.

### *V*_max_ as a Proxy of Enzyme Concentration

To simplify the combinatorial designs used in this study, the focus is on changing the concentration of the *N* pathway enzymes by changing the *V*_max_ of the enzyme in the kinetic model. For each enzyme, the maximum flux is given by *V*_max_ = *k*_cat_*[*E*], where *k*_cat_ is the turnover rate of the substrate by the respective enzyme and [*E*] is the enzyme concentration. As *k*_cat_ is a constant for each gene variant, it follows that modulation of the enzyme concentration by the relative gene up-/downregulation results in a proportional change in the *V*_max_ (eq 1)
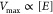
1

### Combinatorial Design Scenarios

To model combinatorial designs, two assumptions are made. First, enzyme levels can be effectively manipulated using some combinatorial DNA library in the range of [0.25–4]. Second, the order of strengths for these DNA elements is known *a priori*. In real metabolic engineering, this prior information could, for example, be predicted using a promoter strength prediction algorithm.^40,54^ Enzyme levels are represented relative to the enzyme concentration of the initial strain. For example, when in a metabolic engineering process, *N* enzymes of a pathway are considered, a twofold upregulation in the second enzyme and twofold downregulation in the fourth enzyme would be encoded as (eq 2)
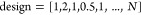
2

From the combinatorial design space containing *P*^*N*^ designs, only a small subset can be experimentally probed. In the equal sampling scenario, we choose each enzyme level with equal probability and assemble the design.^22^ To mimic the effect of biases in the building of strains (*e.g.*, due to experimental limitations), we define two alternative sampling scenarios. The strong effect bias sampling scenario means that stronger enzyme levels with respect to the initial strain are assigned higher probabilities to be sampled. Conversely, the weak effect bias sampling scenario means that we assign a higher probability to enzyme levels closer to the initial strain enzyme level.

### Machine Learning Methods

All models are trained on the three scenarios. The features are the enzymes, where values indicate the enzyme level with respect to the initial strain. Strain designs are encoded as described in the previous section and are used as instances. The target variable is the product flux through enzyme G. For the linear methods, we chose elastic-net and linear support vector machine (linear SVM) regressor. For the nonlinear models, we chose a random forest (RF) regressor, GBR, feed-forward neural network (NN), support vector regressor, and K-NN regressor.^55^

Neural networks, RF regressor, and GBR require hyperparameter optimization in order to prevent overfitting. Hyperparameters were chosen by using Bayesian hyperparameter optimization through a fivefold cross-validation on the training set, using 20 iterations. For this, *scikit-optimize* was used.^56^

### Model Validation

Model performance was evaluated based on two metrics: how well machine learning methods predict unseen designs from the full combinatorial set and the performance in predicting the top 100 highest-producing strains. The Pearson correlation coefficient (*R*^2^) between the predicted *versus* simulated steady-state flux was used to address the general prediction performance. For the performance in finding the global optimum, the fluxes are predicted based on the trained model and the set of the top 100 best-producing predicted designs are compared to the actual top 100 best-producing strains from the simulated combinatorial design space (eq 3)
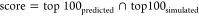
3

As the sampling scenarios are probabilistic in nature, we perform the training set scenario and test set simulation 20 times to estimate the mean and variance *R*^2^.

### Noise Models

Metabolic networks are inherently stochastic, and measurement errors in determining metabolite concentrations and reaction fluxes exist. Two different noise percentages were chosen for testing the robustness of machine learning models to noise. Thus, after the mutant is simulated and compared with respect to the wild-type, noise is added by drawing a value from a normal distribution for a homoscedastic case and heteroscedastic case (σ = 0.04 and 0.15).

### Design–Build–Test–Learn Cycles

Machine learning models were trained on a small subset of designs that were sampled from an initial sampling scenario, as described in the preceding section. After training, models are used to predict the product flux of the combinatorial design space. We developed the following algorithm to recommend designs for the next round based on the machine learning model predictions.

### Recommending New Designs for the Next Cycle

A trained model is used to predict all possible combinatorial designs which, for a pathway with *N* enzymes and with each enzyme having, for example, *P* enzyme levels (that might be achieved by a set of promoters), results in *P*^*N*^ predictions. The strain designs are sorted in ascending order by their predicted flux. A threshold λ* is introduced, which is defined between zero and the maximum predicted flux (0 ≤ λ* ≤ *y*_max_). The threshold λ* is increased from zero to *y*_max_ given a predefined number of evaluations. At each evaluation, only combinatorial designs where the predicted flux is higher than λ* are considered (*y*_pred_ ≥ λ*). For each enzyme, the frequency of enzyme levels is counted and normalized by the total number of designs where *y*_pred_ ≥ λ*.

When λ* = 0, the frequency of each enzyme level is given by *p* = 1/*P*. This is because all combinatorial strain designs have a product flux higher than zero, and therefore, each enzyme level is equiprobable. As λ* is increased, the statistical contribution of each enzyme level to the remaining part of the combinatorial design space can be followed. Promoters that have no significant contribution to higher product flux are expected to be underrepresented.

To transform the threshold plot to a distribution that can be used for sampling new strain designs, the AUC for each enzyme level is determined and normalized. This results in a probability distribution for each considered enzyme as a machine learning-guided way to sample new designs. Finally, we retrain the machine learning model in the next DBTL cycle, also including data from past cycles.^22^

### DBTL Cycle Strategies

Three DBTL cycle strategies were compared for the four best-performing machine learning algorithms. For a fair comparison, at the most, 250 strains are built over multiple cycles.

The first scenario is one where for each DBTL cycle round, an equal amount of strains are built. The second scenario consists of building many strains in the first cycle and then decreasing for each round. Finally, a reversed scenario is defined, where we start with building only few strains, and then increase (Table 3). Each metabolic engineering experiment consisting of five cycles is initialized by an equal sampling scenario of enzyme levels. 15% homoscedastic noise is added to each measurement, and each scenario is performed 30 times. To assess the performance of the DBTL cycle strategies, the top 100 prediction scores and *R*^2^ are reported to give a general impression of the performance of each strategy. Additional performance measures are reported in the Supporting Information.

**Table 3:** DBTL Cycle Scenarios^a^

	cycle 1	cycle 2	cycle 3	cycle 4	cycle 5
scenario 1	50	50	50	50	50
scenario 2	150	50	25	25	
scenario 3	25	25	50	150	

a 250 strains were built over multiple rounds.

## Supporting Information Available

DBTL cycle strategies analysis (XLSX)
DBTL cycle runs for the three scenarios in the main text for gradient boosting, RF, NN, and linear support vector machine, as well as some other performance indicators; additional details on the parameterization of the implemented pathway; results on the machine learning performance; results on the effect of noise on the predictive performance; details regarding the recommendation algorithm, as well as a comparison to the ART; and additional results on the sampling scenarios (PDF)
