# Supplementary material for: Simulated Design–Build–Test–Learn Cycles for Consistent Comparison of Machine Learning Methods in Metabolic Engineering
Source: ACS Synth Biol. 2023 Aug 24;12(9):2588–99. doi: 10.1021/acssynbio.3c00186 (PMC10510747; doi:10.1021/acssynbio.3c00186)
Supplement: Supplementary file 2 — sb3c00186_si_002.pdf [file sb3c00186_si_002.pdf]

Simulated Design-Build-Test-Learn Cycles for Consistent Comparison of Machine Learning Methods in Metabolic Engineering

Supporting Information

Author 1: Paul van Lent

Delft Bioinformatics Lab, Delft University of Technology Van Mourik, 2628 XE Delft, The Netherlands

Author 2: Joep Schmitz

DSM Science and Innovation, Biodata and Translational Sciences, Delft

Author 3: Thomas Abeel

Delft Bioinformatics Lab, Delft University of Technology Van Mourik, 2628 XE Delft, The Netherlands

Infectious Disease and Microbiome Program, Broad Institute of MIT and Harvard, Cambridge, MA 02142, USA (United States of America)

Email: t.abeel@tudelft.nl

Phone number: +31 15 27 85114

Synthetic Pathway: Thermodynamic flux analysis constraints

We included a synthetic pathway with symbolic metabolites and reactions that was inspired on a real-world pathway with thermodynamics. The pathway resembles the Shikimate pathway, which is an important pathway for many aromatic precursors. It draws from the two substrates phosphoenolpyruvate and erythrose-4-phosphate and uses ATP and NADPH [1]. The focus of our study was not to generate the best kinetic model, which would require extensive validation and testing of predictions made by the model. Rather, we aimed to establish a way to make kinetic models with desired properties that can be semi-automated to generate more than one pathway optimization problem for more elaborate validation of optimization strategies in future work [2,3]**.** For the pathway used in this study, all the modelling constraints used can be found in the Python code that is provided on AbeelLab/simulated-dbtl (github.com). The most important properties are reported in the main text, and we further elaborate on the metabolite concentration bounds.

Table S1:Metabolite that were included in the synthetic pathway. Seed IDs are used for adding the thermodynamic constraints on the flux directionality profile. For some other constraints that were used before thermodynamic flux analysis was performed, the SeedID values were already in the model. The concentration bounds are used for further constraining the solution space and were mostly taken from SABIORK[4]. If the concentration could not be found, we assumed that the concentration was bound between 0.00001 and 0.001 mol. The full parameterization can also be found in the file Synthetic_PathwayA.ipynb

**Metabolite name**	**Seed ID**	**Concentration**
A_c	cpd02857	[0.000340*0.8, 0. 000340*1.2] mol
B_c	cpd00699	-
C_c	cpd01716	-

D_c	cpd00383	-
E_c	cpd02030	-
F_c	cpd00932	-
G_c	cpd00216	[0.000045*0.8,0.000045*1.2] mol
Glc-D_e	Already in model	[0.056*0.8,0.056*1.2] mol
Pi_e	Already in model	[0.001*0.8,0.001*1.2] mol
Co2_e	Already in model	[1e-7*0.8,1e-7*1.2] mol
O2	Already in model	[0.062*0.8,0.062*1.2] mol

Synthetic Pathway: ORACLE parameter sampling additional constraints

Additional constraints used for parameterization of the kinetic model are reported here and can also be found in the python notebook **Synthetic_PathwayA.ipynb.** These constraints were used from the tutorials provided in the SkiMPY package, although we did not at all as observed that it could lead to poor biomass growth in the batch fermentation simulation. The following additional constraints were used:

1. Glucose transporter vmax is around 10 mmol/gDW/h
2. The acetate transporter is unsaturated
3. ATP maintenance is saturated

Using ORACLE sampling, the following kinetic parameters were determined for each reaction (Table S2).

Table S2: Kinetic parameters that were found using ORACLE sampling. ORACLE sampling finds large sets of kinetic parameters that are consistent with the steady-state flux defined by thermodynamic flux balance analysis. We chose a parameter set where the dynamics were stable and the timescales of the dynamics when perturbing a parameter are within the timescales of one cell-cycle (see reference for a more elaborate explanation of what is considered physiologically relevant in ORACLE) [5].

**Reaction**	Km substrate 1 (µmol)	Km substrate 2 (µmol)	Vmax (µmol/s)	K equilibrium	Km product 1	Km product 2
A	1613.07	20.03	15193.97			
B	814.68		31472.31			
C	307.26		14893.06	7799269.43	3946.54	
D	42725.09	192.59	5809120.89	30.51	227.53	0.856
E	1695.23	3505.46	66347.89	74208.67	26345.98	1706.60
F	1252.32	769.28	98247.12	0.048	6139.34	417.49
G	3024.44		124061.73	672620138 10853	74.45	2137.69

Training set sizes larger than 1000

We increased the training set sizes of Figure 4 to larger values, which would normally not be encountered in Metabolic Engineering, to test whether certain algorithms saturate. We noticed that Stochastic Gradient Descent saturates in predictive performance, even though for the top 100 prediction it outperforms the nonlinear methods when limited in samples (N=50). We observe that

Random Forest and Gradient Boosting performs well for the R^2. For the four algorithms, we performed Bayesian hyperparameter optimization.

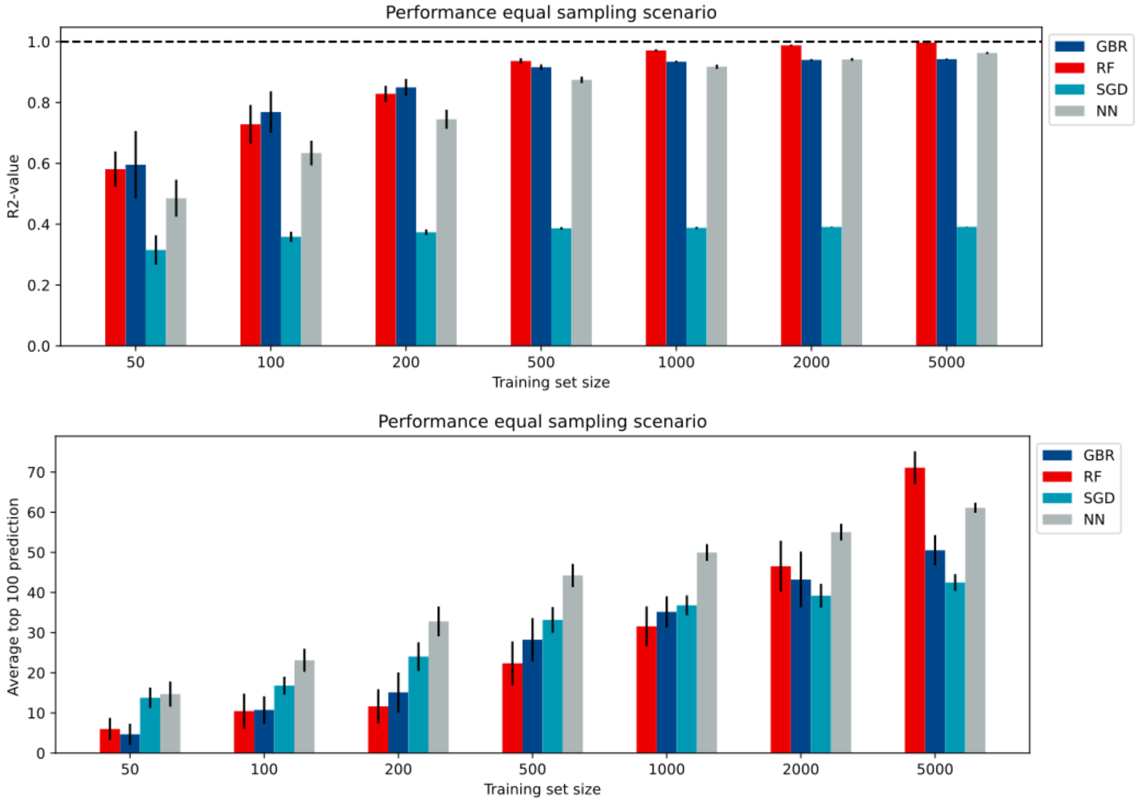

Figure S1: Performance of the four algorithms when trained on set sizes of up to 5000 samples. The nonlinear ensemble methods are the best performers, although Neural Networks tend to also the top 100 quite well.

Effect of noise on predictive performance

We tested homoscedastic and heteroscedastic noise with 4% and 15% noise percentages. Homoscedastic noise is independent of the mean experimental value (Figure S2A, B). Heteroscedastic noise has a mean dependent standard deviation (Figure S2C, D). To assess the effect of noise on predictive performance, we compared the four different noise models (homoscedastic 4% noise, homoscedastic 15% noise, heteroscedastic 4% noise, heteroscedastic 15% noise) to the no noise scenario. This was performed for the four best performing algorithms (linear Support Vector Machine, Gradient Boosting, Random Forest, and Neural Network). Each model was trained with Bayesian hyperparameter optimization twenty times and the top 100 prediction and R^2 value was calculated (see *Methods*). We tested for 50 samples and 200 samples and performed a multiple hypothesis testing corrected t-test (Bonferroni). The results are summarised in Table S3. Over the 20 runs, we do not observe significant effects on the R^2. Sometimes differences are observed in the top 100 prediction for the 4% heteroscedastic noise model (Table S4).

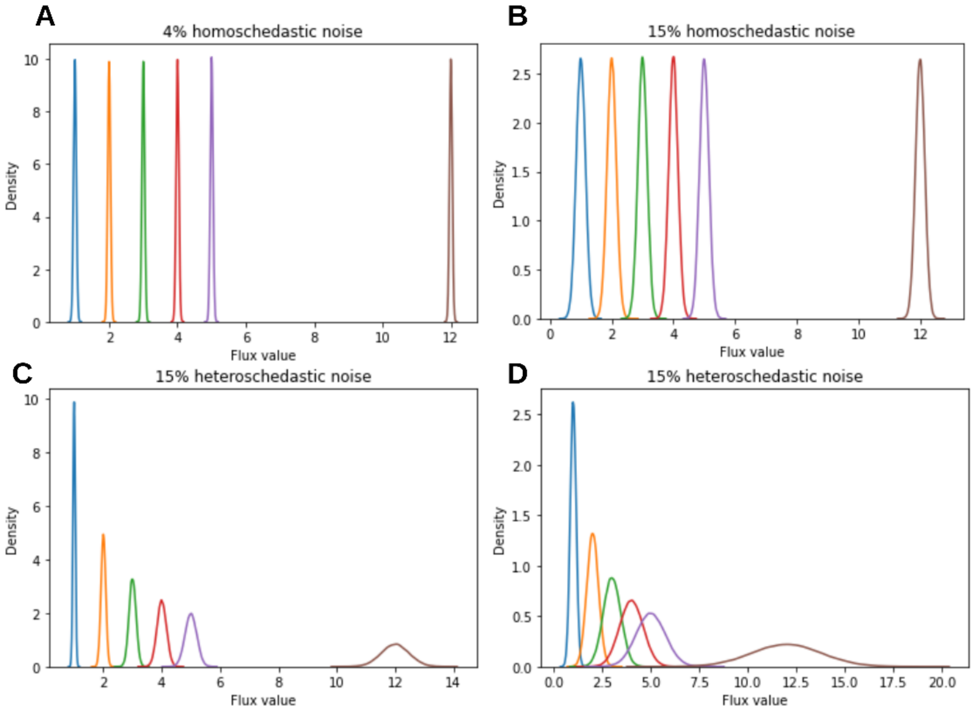

Figure S2: Two different noise models with 4% and 15% noise. The level of noise for the heteroscedastic case is dependent on the experimentally determined mean value (I.e., variance increases with increasing mean).

Table S3: Results for all methods for the top R2. We tested for each algorithm between all noise models and no noise, which results in 10 tests for each algorithm. In the table, we report whether significant differences between a noise model and the no noise scenario were observed. N.s. means non-significant differences

**Algorithm**	**N=50**	**N=200**
**Neural Network**	n.s.	n.s.
**Gradient Boosting**	n.s.	n.s.
**Random Forest**	n.s.	n.s.
**Linear SVM**	n.s.	n.s.

Table S4: Results for all methods for the top 100 prediction. Here we do sometimes observe some difference between no noise and the heteroscedastic model. The significantly different pairwise tests are reported in the table. N.s. means non-significant differences.

**Algorithm**	**N=50**	**N=200**
**Neural Network**	Heteroscedastic noise (4%)	n.s.
**Gradient Boosting**	n.s.	Heteroscedastic noise (4%)
**Random Forest**	n.s.	n.s.
**Linear SVM**	Heteroscedastic noise (4%)	n.s.

The automated recommendation algorithm and feature importance

All code can be found in the comb_sampling.py file. The two most essential functions are:

1. scan_combinatorial_space
2. generate_frequency matrix
3. find_set_designs (hardcoded version)

Here, we show an example of the predicted frequencies along the designs space for enzyme A (N=100).

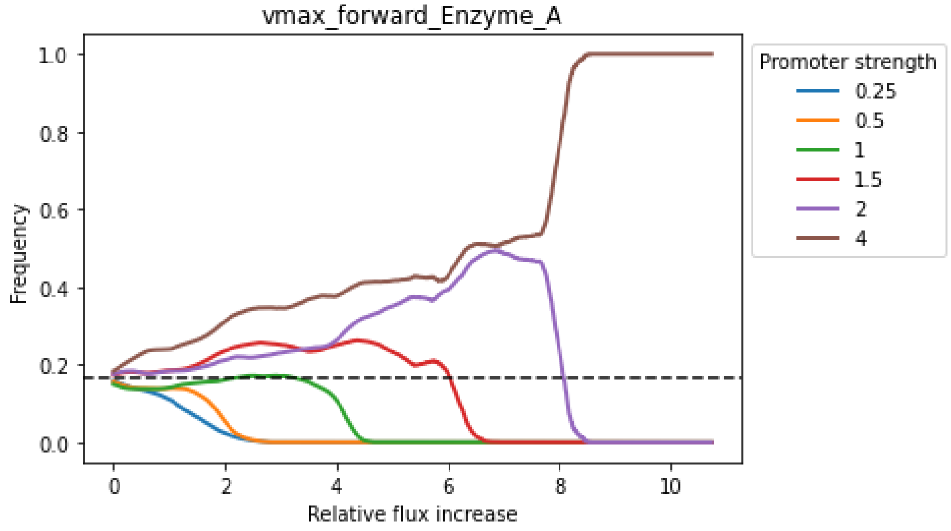

Figure S3: Frequency of promoters for enzyme A as a function of a threshold (>=J).

Then, we take the AUC for all the promoter strengths and normalize. Doing this for every enzyme we get a probability distribution to sample for the next DBTL cycle.

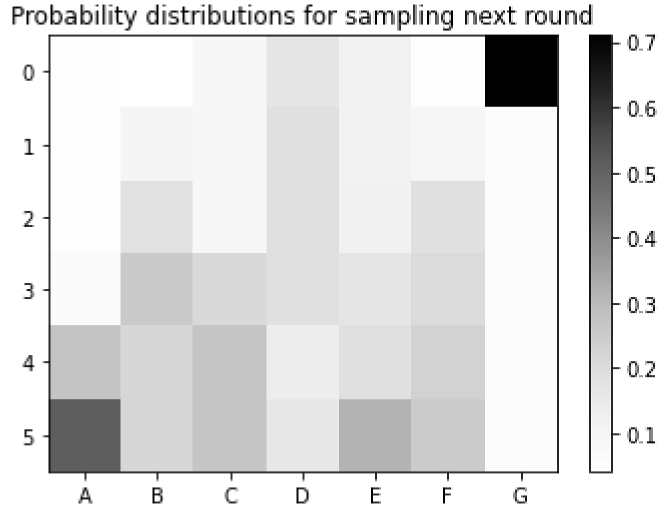

Figure S4: Estimated probability distribution to sample promoters from for next DBTL cycle round.

We can also calculate the entropy of the probability distribution for each enzyme, which gives an indication of how important each enzyme (feature). As can be seen, enzyme G, C, and A are the most important enzymes because their entropy decreases more rapidly than others. A decrease in entropy indicates that the probability distribution is more biased (favours certain promoter strengths).

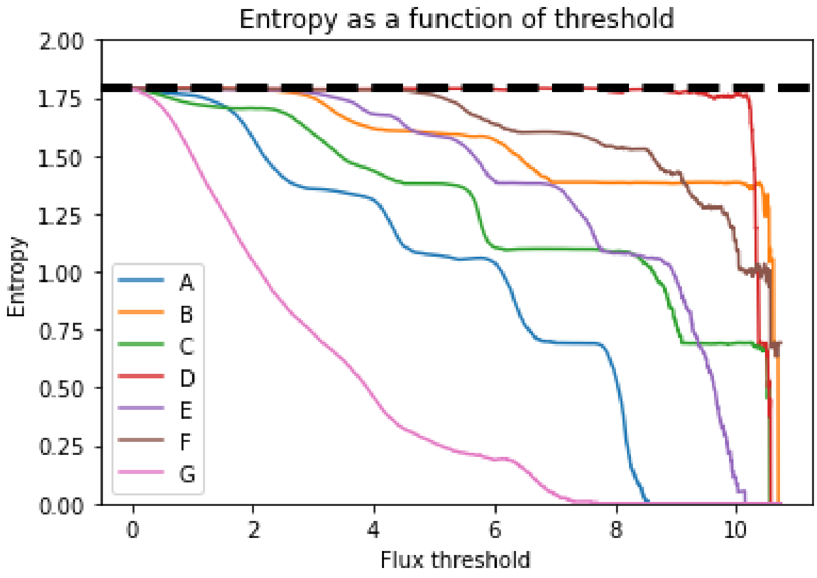

Figure S5: estimated entropies of the features in the learned space. As can be seen, Enzyme A, C, G have the largest contributions to high flux. If the entropy is 0, then this means that one promoter is completely fixed in that region where the flux is higher than that value.

Example run of 5 cycles: GBR with 25 designs, 15% homoscedastic noise

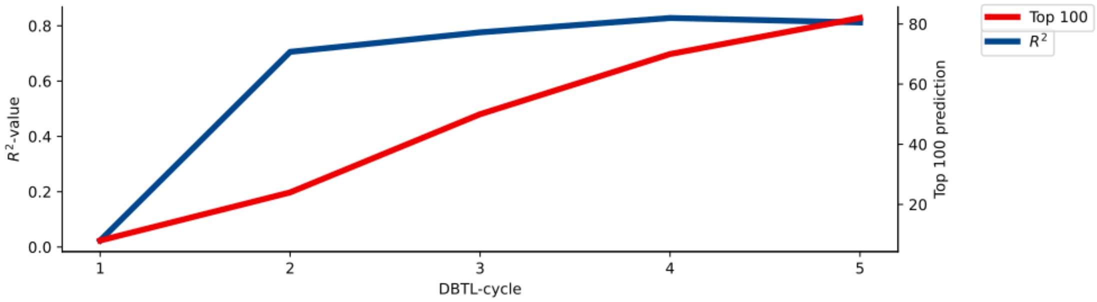

Figure S6: One example run of the fully automated recommendation algorithm. Every cycle 25 designs were built, and the top 100 and R^2 are calculated to follow the performance of the optimization. We noticed that in this example run the best performing strain is in the predicted top 100 from round three onwards.

Statistical analysis of the optimality of designs

Important in the implementation of DBTL cycles is the initial sampling. In this paper we have mostly chosen random sampling scenarios (each promoter is chosen with equal probability), but many other initial sampling strategies exist. For example, the Automated Recommendation Tool uses Latin Hypercube Sampling, which outperforms random sampling slightly (see Figure S8) [6]. The effects of the initial sampling on model performance and optimization of DBTL cycles can be easily assessed using simulated DBTL cycles, both empirically and using optimality criteria such as D-optimality[7].

D-optimality of initial sampling scenario's

Given that we have a linear model consisting of main-effects and interactions between features. Specific to our problem that has seven perturbed enzymes, the linear model could be written down as follows:

$$Y = \beta_0 + \sum_{i=1}^{7} \beta_i \, x_i + \sum_{i=1}^{6} \sum_{j=i+1}^{7} \beta_{ij} \, x_i \, x_j + error$$

Here Y product flux through reaction G, β_0 is an intercept term, β_i are the main factor parameters, and β_{ij} are the interaction parameters. The number of parameters of a main-factor and interaction model for this case has $p = \frac{(k+1)(k+2)}{2} = 36$ parameter terms. The model can be written in matrix notation as:

$$Y = X\beta + error$$

Where Y is the n-dimensional response vector, and X is the $n * p$ model matrix[8]. Assuming that all n random errors are independent and identically distributed, the best linear unbiased estimator for the parameter vector β is given by the ordinary least squares estimator:

$$\beta = (X^T X)^{-1} X^T Y$$

The thing we are interested in here is the inverse of the variance-covariance matrix, which is also known as the Fisher information matrix:

$$\sigma^{-2}(X^T X)$$

A design set is referred to D-optimal when it maximizes the determinant of the Fisher information matrix. Such an optimality criteria can be used to measure the efficiency of a sampling scenario with respect to an optimal (orthogonal) design by the equation:

$$D_{eff} = \left[\frac{\det(X^T X)}{\det(X_D^T X_D)}\right]^{1/p}$$

Where X_D is the D-optimal model matrix. When $D_{efficiency}$ is close to one, the design is considered optimal given this criterion. To show how this might be used to assess sampling scenarios, we show here the optimality criterion for a Latin Hypercube sampling and Random Sampling compared to an optimal design strategy, implemented using the *dexpy*-package.

Figure S7A shows the D-efficiency for random sampling and Latin Hypercube sampling (LHS) with respect to the D-efficiency of a D-optimal design scenario for 50 and 200 designs. A value close to one would indicate that the sampling strategy has a similar D- efficiency to a D-optimal design. Both LHS and random sampling have a low D-optimality compared to a D-optimal design, as expected. Also, LHS has a lower variance in D-efficiency than random sampling, which is also expected due to the constraints that are imposed in the Latin Hypercube algorithm. Interestingly, when considering model performance in terms of the Pearson Correlation Coefficient (R^2), we observe that LHS and random sampling seem to outperform D-optimal designs. While this observation is not completely clear from the top 100 prediction, this could suggest that D-optimality is not necessarily an appropriate optimality criterion when the modelling problem is nonlinear, as here a linear model with interacting factors was assumed. Further work on this matter would be required to make conclusive remarks on this.

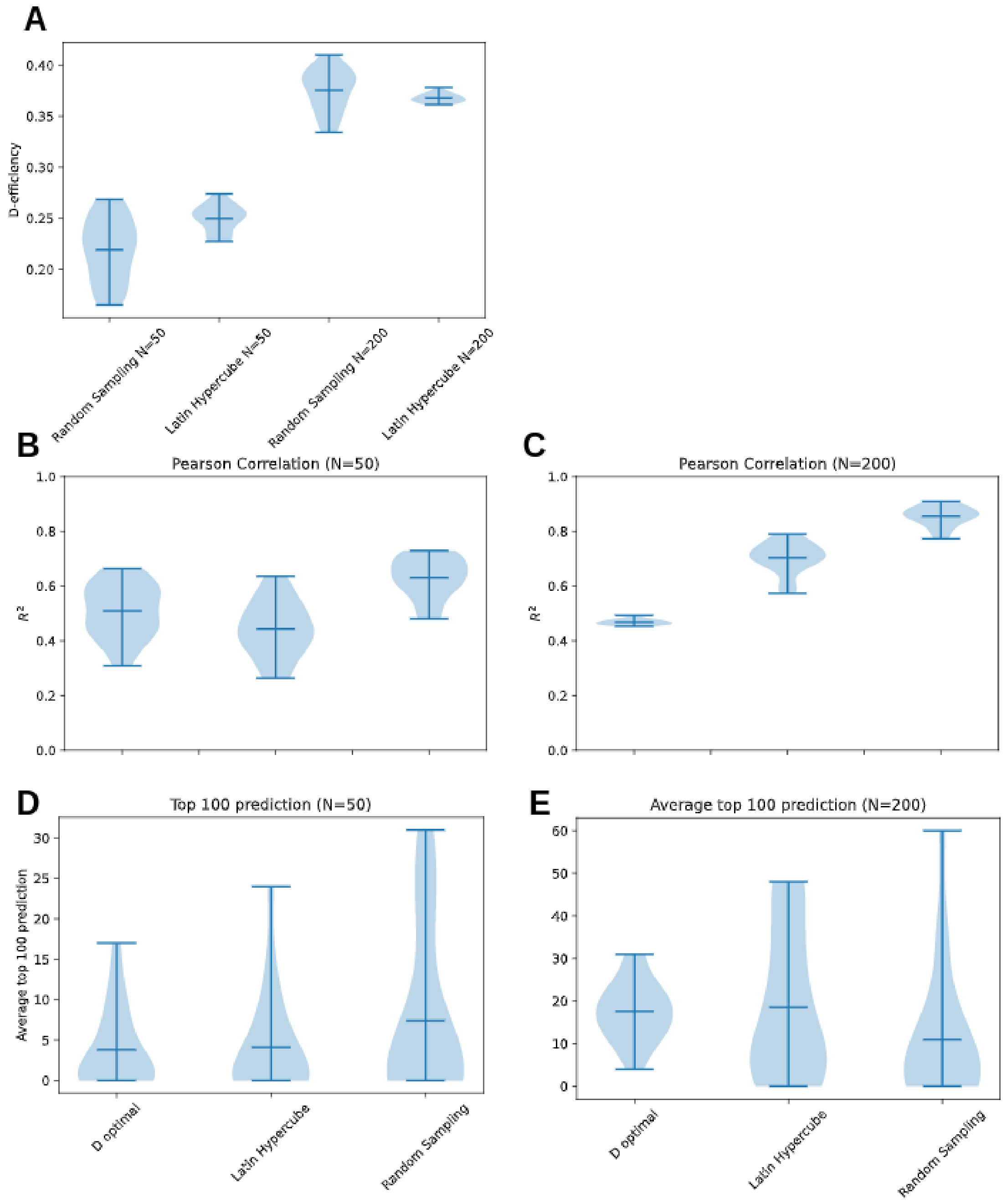

Figure S7: ***Comparing sampling scenarios based on D-efficiency and model performance.*** *A) D-efficiency of Latin Hypercube sampling and random sampling for N=50 and N=200 with respect to a D-optimal design scenario. When the number of samples is higher, D-efficiency is higher. B, C) Model performance for D-optimal designs, Latin Hypercube sampling, and random sampling (30 runs). Overall, no significant difference was observed between the three sampling strategies when the number of samples is small (50 strains). However, if the number of strains built is increased to 200, Latin Hypercube sampling and random sampling outperform D-optimal designs. D, E) For the top 100 prediction, there is not really a pattern observed, or a significant difference between the three methods. Random sampling has a slightly larger variance for the different runs, indicating that the other methods are more stable.*

DBTL cycle scenario simulation for the four best performing algorithms.

Results on the performance of algorithms over multiple DBTL cycles are reported in DBTL Cycle strategies analysis.xlsx: **Table S5**

The Automated Recommendation Tool on simulated data

One notable recommendation algorithm is the Automated Recommendation Tool (ART)[9]. Simulated Design-Build-Test-Learn cycles can be used to benchmark methods easily. Here, we highlight how this can be used to evaluate the performance of different recommendation algorithms. ART outputs a list of a predefined number of strains you want to build.

Here, five DBTL cycles were performed for ten runs, with an exploration/exploitation (α) parameter that changes over the course of the five cycles. For this example, the α-parameter was set to 0.8, 0.6,0.4,0.2 for DBTL round 2,3,4,5, respectively. ART recommendations (blue) were compared to the method described in this paper (orange) by their best recommended strain compared to the best performing strain in the full combinatorial space. As can be seen, ART performs better than the Gradient Boosting recommendation strategy described in this paper. We hypothesize that this can be attributed to the α-parameter that ART uses, as well as a slightly higher initial recommendation strategy (Latin Hypercube sampling). Other settings might lead to different results on performance. This would have to be further tested using simulated DBTL cycles.

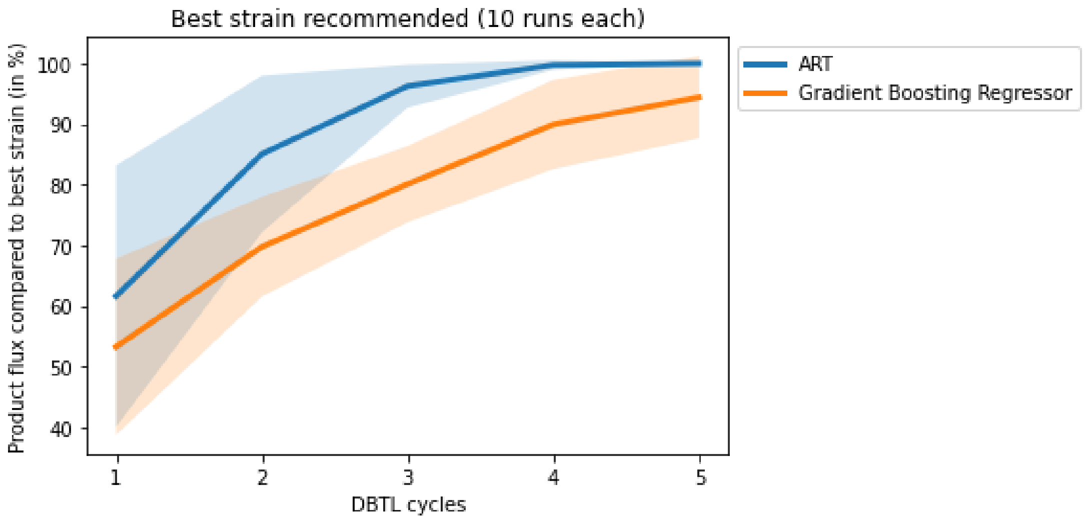

Figure S8: ART is compared to the recommendation algorithm described in this study, which in this case uses Gradient Boosting. The ART algorithm is performing better than the Gradient Boosting based recommendation algorithm in this example.

Additional objectives for strain optimization

The focus in this paper has been on metabolic flux optimization of a synthetic pathway. In some cases, this might not be the only factor a metabolic engineer wants to consider. Factors like biomass growth, the accumulation of toxic intermediates, or genetic stability might need to be accounted for when optimizing strains. In this regard, metabolic engineering can be considered a multi-objective optimization problem, and one can then consider tools for optimization as described in[10]. One

simple solution for multi-objective optimization is that you aggregate the optimization targets (e.g., biomass, product formation) in one scalar value that is used as the target variable.

Figure S8 shows the biomass growth of the initial strain and the best producing strain in a multi-species batch reactor [2]. The best producing strain has less biomass growth. We therefore assume that biomass here does not have a drastic effect on the producing capabilities of the optimized strain. If the pathway considered here would have toxic intermediate compounds that have an inhibiting effect on biomass growth, this could be considered by doing multi-objective optimization[10].

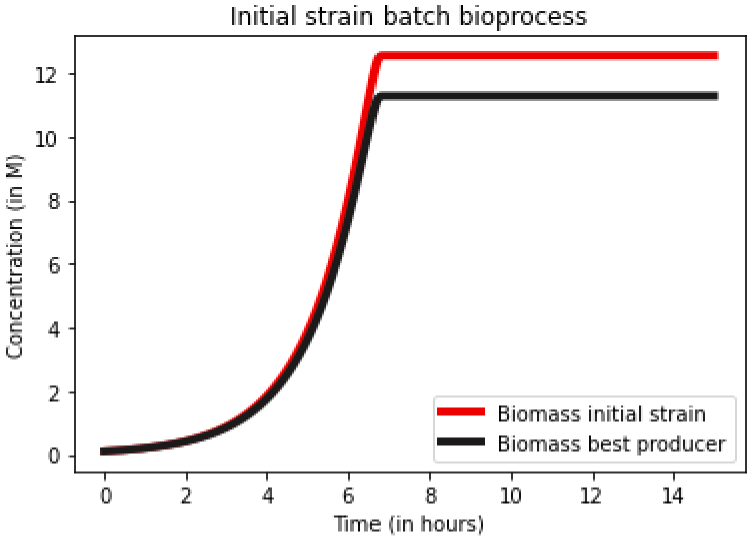

Figure S9: A multispecies batch bioprocess implemented using SKiMpy [2]. The initial strain produces more biomass than the best producer when computing for a glucose feed. However, the best producer produces >10 times more flux towards product G then the initial strain. When performing multi-objective optimization, the learned target variable could be a composition of both Biomass and flux through product G, weighted by their relative importance.

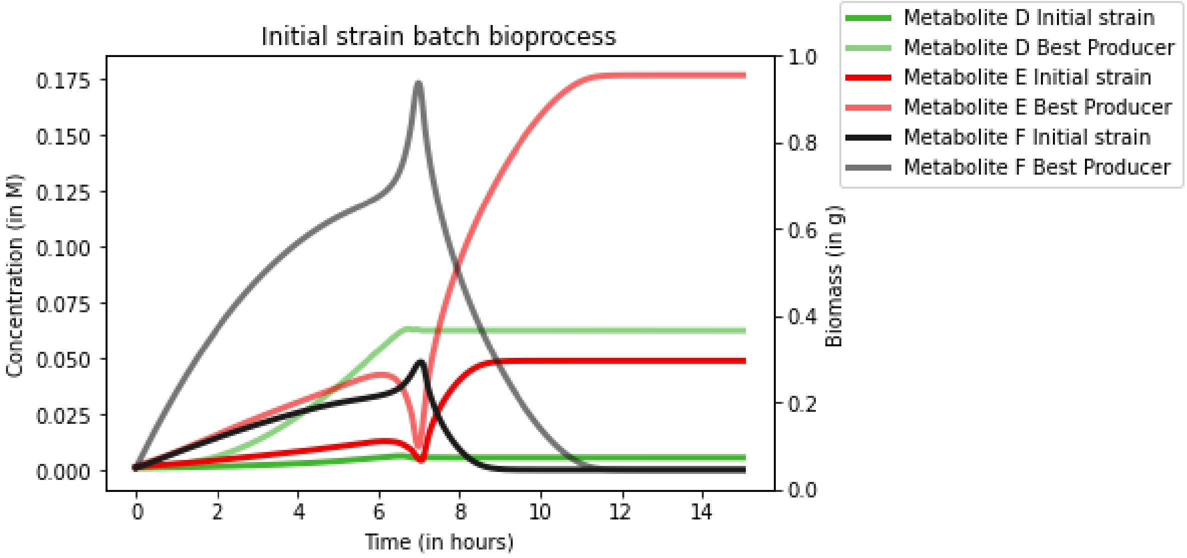

Figure S10: Intracellular metabolite concentrations for the initial strain and the best producing strain. The best producing strain has very high levels of intracellular concentrations. If during strain optimization some metabolites are considered toxic, this could be accounted for by giving a negative weight to high concentrations in the multi-objective optimization problem[10].

References

1. Averesch NJH, Krömer JO. Metabolic engineering of the shikimate pathway for production of aromatics and derived compounds-Present and future strain construction strategies. *Front Bioeng Biotechnol*. 2018;6(MAR):32. doi:10.3389/FBIOE.2018.00032/BIBTEX
2. Weilandt DR, Salvy P, Masid M, et al. Symbolic Kinetic Models in Python (SKiMpy): Intuitive modeling of large-scale biological kinetic models. *bioRxiv*. Published online January 20, 2022:2022.01.17.476618. doi:10.1101/2022.01.17.476618
3. Miskovic L, Hatzimanikatis V. Production of biofuels and biochemicals: in need of an ORACLE. *Trends Biotechnol*. 2010;28(8):391-397. doi:10.1016/J.TIBTECH.2010.05.003
4. Wittig U, Rey M, Weidemann A, … RK-N acids, 2018 undefined. SABIO-RK: an updated resource for manually curated biochemical reaction kinetics. *academic.oup.com*. Accessed February 11, 2023. https://academic.oup.com/nar/article-abstract/46/D1/D656/4577570
5. Choudhury S, Moret M, Salvy P, Weilandt D, Hatzimanikatis V, Miskovic L. Reconstructing Kinetic Models for Dynamical Studies of Metabolism using Generative Adversarial Networks. *Nat Mach Intell 2022 48*. 2022;4(8):710-719. doi:10.1038/s42256-022-00519-y
6. McKay MD, Beckman RJ, Conover WJ. A comparison of three methods for selecting values of input variables in the analysis of output from a computer code. *Technometrics*. 2000;42(1):55-61. doi:10.1080/00401706.2000.10485979
7. Carbonell P, Faulon JL, Breitling R. Efficient learning in metabolic pathway designs through optimal assembling. *IFAC-PapersOnLine*. 2019;52(26):7-12. doi:10.1016/J.IFACOL.2019.12.228
8. Jones B, Allen-Moyer K, Goos P. A-optimal versus D-optimal design of screening experiments. *https://doi.org/101080/0022406520201757391*. 2020;53(4):369-382. doi:10.1080/00224065.2020.1757391
9. Radivojević T, Costello Z, Workman K, Garcia Martin H. A machine learning Automated Recommendation Tool for synthetic biology. *Nat Commun*. Published online 2020. doi:10.1038/s41467-020-18008-4
10. Gunantara N. A review of multi-objective optimization: Methods and its applications. *http://www.editorialmanager.com/cogenteng*. 2018;5(1):1-16. doi:10.1080/23311916.2018.1502242
